# Underwater Acoustic Target Recognition Based on Depthwise Separable Convolution Neural Networks

**DOI:** 10.3390/s21041429

**Published:** 2021-02-18

**Authors:** Gang Hu, Kejun Wang, Liangliang Liu

**Affiliations:** 1 College of Automation, Harbin Engineering University, Harbin 150001, China; hugang@hrbeu.edu.cn (G.H.); liuliangliang@hrbeu.edu.cn (L.L.); 2 College of Business, Anshan Normal University, Anshan 114007, China

**Keywords:** underwater acoustic target, ship radiated noise, deep learning, depthwise separable convolution, dilated convolution

## Abstract

Facing the complex marine environment, it is extremely challenging to conduct underwater acoustic target feature extraction and recognition using ship-radiated noise. In this paper, firstly, taking the one-dimensional time-domain raw signal of the ship as the input of the model, a new deep neural network model for underwater target recognition is proposed. Depthwise separable convolution and time-dilated convolution are used for passive underwater acoustic target recognition for the first time. The proposed model realizes automatic feature extraction from the raw data of ship radiated noise and temporal attention in the process of underwater target recognition. Secondly, the measured data are used to evaluate the model, and cluster analysis and visualization analysis are performed based on the features extracted from the model. The results show that the features extracted from the model have good characteristics of intra-class aggregation and inter-class separation. Furthermore, the cross-folding model is used to verify that there is no overfitting in the model, which improves the generalization ability of the model. Finally, the model is compared with traditional underwater acoustic target recognition, and its accuracy is significantly improved by 6.8%.

## 1. Introduction

Underwater acoustic target recognition technology is used to analyze ship radiated noise received by sonar and to judge the classification of the target [1,2], which has important economic and military value. Because of the complex marine environment and application of acoustic stealth technology, underwater acoustic target recognition has always been an internationally recognized problem. Traditional underwater acoustic target recognition methods based on ship radiated noise classify ship types by using artificially designed features and shallow classifiers, focusing on feature extraction and the development of nonlinear classifiers [3,4,5,6,7,8]. The features of artificially designed ship-radiated noise include waveform [9], spectrum [10], and wavelet [11], etc., which are dependent on expert knowledge and prior knowledge and have weak generalization ability. Shallow classifiers such as support vector machine (SVM) [12] and the shallow neural network classifier [13] have weak fitting and generalization abilities when dealing with complex and large numbers of samples. Generally speaking, classifier design and feature extraction are conducted independently, which may lead to the design of the feature not being suitable for the classification task. For example, in the classification model based on auditory features, auditory filter banks designed based on perceptual evidence tend to focus only on the property of signal description rather than the purpose of classification [14,15].

The human brain has strong abilities in perception, reasoning, induction, learning, etc. Inspired by the human neural structure and brain information processing mechanism, researchers proposed deep neural networks (DNNs) which processed information and decision-making in a similar way to brain [16,17]. Due to the emergence of deep learning technology, a complete deep learning model can not only realize the mathematical modeling for the original signals but also predict targets. These findings of auditory system research show that the acoustic signals of an auditory system in the time domain can be decomposed into frequency components; different regions of the auditory system perceive information of different frequency components; the brain uses information from all these regions to analyze and classify acoustic signals. In addition, research on the plasticity of the auditory cortex has demonstrated that the adult brain can be reshaped in appropriate environments [18]. 

The language model method has been widely used in natural language processing [18,19,20]. Drossos et al. applied the language model to detect acoustic events and achieved good effects [21]. The advantage of the language model method was that the input of the recursive neural network (RNN) could be adjusted according to the previous prediction of the classifier, and RNN could be used to process long-term dependencies in temporal information and to model class activities in context so as to learn longer in-class and inter-class time models. When the language model method was used for underwater acoustic target recognition, the performance could be improved by modeling these class dependencies [21]. 

Inspired by auditory perception and the language model, in this paper, a new depthwise separable convolutional neural network for underwater acoustic target recognition is proposed, which consists of a series of depthwise separable convolutions, integration layers, and time-dilated convolutions. The depthwise separable convolutions with variable convolution kernel width are used to decompose original time-domain ship-radiated noise signals into different frequency components, and extract signal features based on auditory perception. Due to the use of a variety of convolution kernels of different sizes, the model can achieve frequency decomposition and feature extraction with a variety of frequency and time precision. Compared with the traditional method of feature extraction based on frequency data, this method solves the contradiction between time precision and frequency precision well, and preserves the phase information of the model input signal to the maximum extent in the process of feature extraction. In the fusion layer, the one-dimensional feature vectors extracted at several consecutive moments are fused to form a two-dimensional feature matrix, which adds time information to the one-dimensional feature vectors. Finally, we use the time-dilated convolution for the modeling of long time attention, which can make full use of the intra-class and inter-class information for underwater acoustic target recognition just like the language model.

The remainder of this paper is organized as follows. In Section 2, we introduce related work in underwater acoustic target feature extraction and recognition. In Section 3, the method is proposed in detail. We describe the evaluation process in Section 4. In Section 5, experimental results are given and discussed. We give our conclusions in the last section.

## 2. Related Work

All existing studies on passive underwater acoustic target feature recognition with deep learning were still in a preliminary stage and focused on theoretical exploration and small-scale experiments. Generally speaking, applications of deep learning should be combined with big data. However, due to the limitations of practical conditions, it was often difficult to collect sufficient data for model training, which greatly limited the performance of deep neural network. Nevertheless, urgent demands still promoted continuous development of deep learning in passive underwater acoustic target recognition. Convolutional Neural Network (CNN) had a variety of applications in passive underwater target recognition because it was suitable for processing the original underwater acoustic signals and could obtain the implicit correlation that is difficult to be found by conventional feature analysis methods to a certain extent [22,23,24,25]. With internal feedback mechanism, RNN can process time-series signals. The audio signal is a typical sequence signal, which is provided with a memory function by RNN through circumferential joints of internal neurons. Therefore, the correlation of acoustic signals in the time dimension can be utilized dynamically. In RNN, there is a typical structure called “Long Short Term Memory” (LSTM) [20], which has been applied to passive underwater target recognition [26,27,28].

In a paper previously published on audio classification, CNN was replaced by depthwise separable (DWS) convolution [29,30]. DWS was a decomposition form of standard convolution, which decomposed a standard convolution into one convolution and one 1 × 1 convolution (called pointwise convolution) [31]. It firstly learned spatial information and then processed to cross-channel mode [32]. This convolutional decomposition for typical CNN resulted in less trainable parameters and memory occupation, and the reduction in computation complexity was $K_{o}^{-1}+{\left(K_{h}\cdot K_{w}\right)}^{-1}$, where $K_{h}$ and $K_{w}$ were the height and width of the CNN kernel, respectively, and $K_{o}$ was the output channel of the CNN [27]. Dilated convolution was considered a method of improving the long-term learning capacity of the CNN [33]. In short, the kernel of the dilated convolution was dilated and there was a distance between two elements. As a result, the kernel of dilated convolution could be used on elements of its input patch with interval *N* (dilation factor), increasing the receptive field of the kernel instead of its parameters [34,35]. The kernel dilation could be used in any combination (for example, dilation in time dimension or feature dimension only) or all combinations of its dimensions. Li et al. provided a method to combine dilated convolution with RNN in audio classification task [36], which clearly focused on the exploration and learning of long-term patterns. Drossos et al. proposed an improved Convolutional Recursive Neural Network (CRNN) structure [31] which used DWS and dilated convolution with dilation in the time dimension only, i.e., time-dilated convolution. With discrete wavelet and time-dilated convolution, this structure had 85% fewer parameters than CRNN, but achieved better performance on typical audio classification datasets. The improvement of the dilated convolution showed that these convolutions had similar functions with RNN and could be used effectively for long-term contextual modeling. 

For human beings, acoustic perception and recognition are accomplished through the auditory system, including the auditory periphery and auditory center. Frequency receptive fields in the auditory center, auditory cortex, auditory midbrain and other structures can adjust the frequency receptive fields and the optimal frequency to complete the learning task [37,38]. These findings about the auditory system indicated that the acoustic time-domain signals could be decomposed according to the frequency components in the auditory system. The decomposition of frequency components could be explained as product filtering for acoustic frequency-domain signals. Since the product of frequency domain signals is equal to the convolution of the time-domain signal [39], the frequency-domain component could be quickly realized by parallel computation of time-domain convolution; different regions of the auditory system perceived different frequency components; the brain collected information of all areas for analysis and for classifying acoustic signals. In addition, studies on the shaping of auditory cortex have shown that the adult brain could be reshaped in an appropriate environment. The auditory experience could change the functions and even structure of the auditory system.

## 3. Deep Convolution Neural Networks

### 3.1. The Structure of the Model

**Figure 1:**
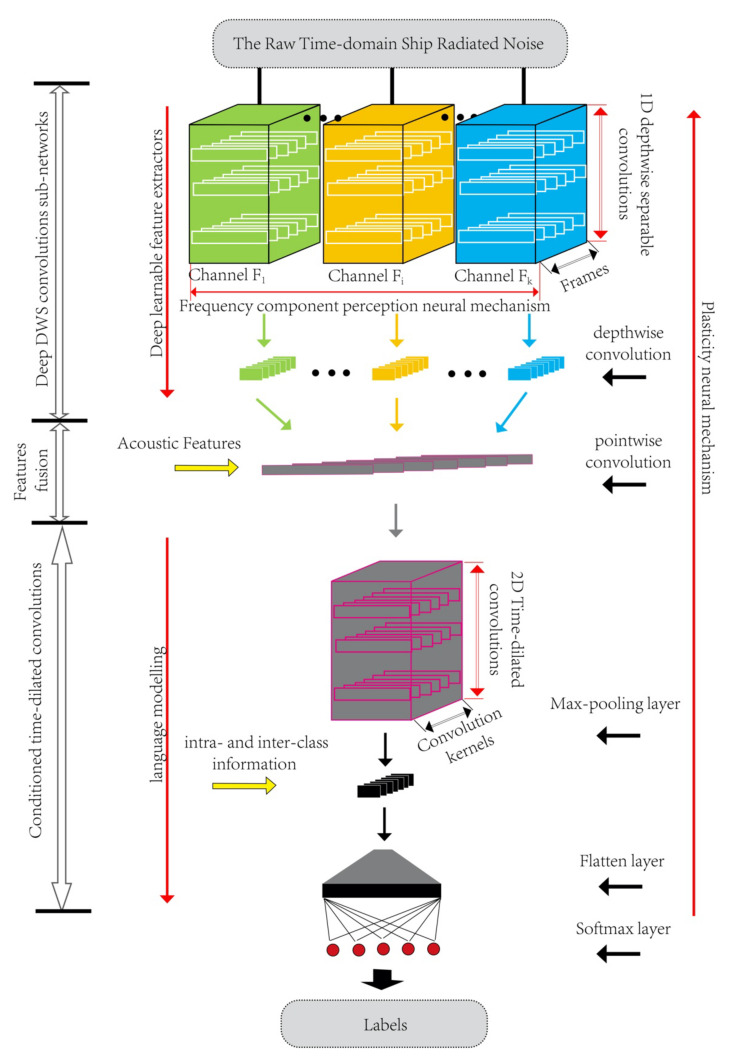
The structure of depthwise separable convolutional neural networks.

We propose a new deep convolution neural network model for feature extraction and classification of ship radiated noise, which includes a series of depthwise separable convolution, fusion layer and time-dilated convolution. The structure of the proposed model is shown in Figure 1.

The model proposed in this paper takes a sequence $X\in {\mathbb{R}}^{T\times N}$ of vectors T as input, and each vector T is composed of original underwater acoustic time-domain data with length N. A learnable feature extractor composed of depthwise separable convolution (DWS) and time-dilated convolution is used as a time pattern recognition The label vector of C classes corresponding to X is $Y=\;\left[y_{1},\cdots ,y_{C}\right]$, where $y_{c}\in \left\{0,1\right\}$, represents whether the input belongs to class *c*. The model output is a vector $\hat{Y}=\;\left[{\hat{y}}_{1},\cdots ,{\hat{y}}_{C}\right]$, where ${\hat{y}}_{c}\in \left[0,1\right]$ represents the prediction classification result of the model for underwater acoustic target when X is input. Inspired by the structure of extracting deep acoustic information of auditory system, we design a series of depthwise separable convolutions, takes the original underwater acoustic data as input and is completed by the one-dimensional depthwise separable convolution neural network. In addition, DWS convolution realizes the frequency decomposition for input signals and extract the features of decomposed signals. 

The feature fusion layer realizes multi-channel feature information fusion by pointwise convolution at every moment. In the feature fusion layer, all one-dimensional acoustic features outputted by DWS filter at all T times are combined and analyzed comprehensively. The combined acoustic features are two-dimensional time mode features at T moments, which can be used as the input of the time-dilated convolution layer. The two-dimensional frequency convolution layer is applied to preserve locality and reduce frequency spectrum change ship-radiated noise. In the time-dilated convolution layer, in order to make use of the intra-class and inter-class activity mode, we adopt a time-dilated convolution like language modeling and use Softmax layer classifier to obtain a prediction probability for each ship of each sample. The classification of ship is taken as the target function, then driven by the original ship-radiated noise signal, and the learning and optimization are carried out in the whole training process. This optimization mechanism reflects the shaping neural mechanism of the auditory system. 

This model can realize decomposition, feature extraction and classification for ship-radiated noise and be used for underwater acoustic target recognition.

### 3.2. Depthwise Separable Convolution

CNN is an artificial neural network signal (ANN) convolution which carries out a series of convolutions for input. The operation in CNN is equivalent to time-domain convolution in a traditional filter [40]. In this paper, multi-layer CNN is designed in each deep filter to realize filtering function, so we define it as a deep convolution filter. Through repeating the above process layer by layer, the multi-layer CNN construct can extract more abstract features from deep structure. However, deeper units may be indirectly connected to all or most of the signals. The receptive field of deep units in a depthwise convolution is larger than that of the shallow unit [41]. The parameters of the depthwise separable convolution filter are randomly initialized and learnt from ship-radiated noise. Driven by the time-domain signals of ship-radiated noise, the frequency decomposition ability of the depthwise separable convolution filter is learnable and adjustable. In addition, larger convolution kernels can contain longer wavelengths, which implies lower frequencies of components and vice versa. Thus, the learned filter is more suitable for the underwater acoustic target recognition task.

In order to learn spatial information and cross-channel information, we do not use the convolution of a single kernel, but the convolution of two different kernels in a series (that is, the output of the first is the input of the second). This decomposition technique is called deep separation convolution (DWN) and has been used in a variety of image processing structures (such as Exception, GoogleLeNet, Inception and MobileNets model). It has been proven that DWS convolution can reduce the number of parameters and improve performance [42,43,44]. DWS convolution consists of depthwise convolution and pointwise convolution. In the deep convolution layer, only one filter is applied per input channel. Pointise convolution is a simple 1 × 1 convolution that is then used to create linear combinations of the depthwise layer outputs. The learnable feature extractor consists of DWS convolution blocks. The lth DWS convolution block obtains output of the previous block as input, i.e., $H_{l-1}\in {\mathbb{R}}^{H_{l-1}^{c}\times H_{l-1}^{h}\times H_{l-1}^{w}}$, where $H_{l-1}^{c}$, $H_{l-1}^{h}$ and$\;H_{l-1}^{w}$ are the number, height and width of channels output by the l−1th DWS convolution block, respectively. $H_{0}=X$ is the input time-domain signal of the ship-radiated noise, and $H_{0}^{c}=1$, $H_{0}^{h}=1$, $H_{0}^{w}=N$. The output of the l^th^ DWS convolution block is $H_{l}\in {\mathbb{R}}^{H_{l}^{c}\times H_{l}^{h}\times H_{l}^{w}}$, where $H_{l}^{c}$, $H_{l}^{h}$ and$\;H_{l}^{w}$ are the number, height and width of channel output by the l^th^ DWS convolution block, respectively. Each DWS convolution block includes one DWS convolution operation, one normalization, one down-sampling and one non-linear function.

**Figure 2:**
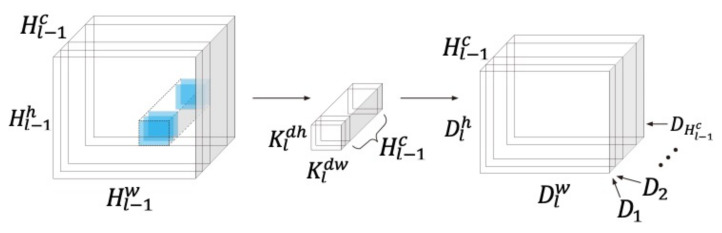
The first step of the depthwise separable convolution: learning spatial information, using $H_{l-1}^{c}$ different kernels $K_{l}$, applied to each $H_{l-1}$.

The operation of DWS convolution itself includes two convolutions, one normalization process and one rectified linear unit (ReLU). The first convolution on the l^th^ DWS convolution block uses $H_{l-1}^{c}$ number of kernels $K_{l}\in {\mathbb{R}}^{K_{l}^{dh}\times K_{l}^{dw}}$ and one bias deviation $a_{l}\in {\mathbb{R}}^{H_{l-1}^{c}}$ to learn spatial information input into $H_{l-1}$. $K_{l}^{dh}$ and $K_{l}^{dw}$ are the height and width of the kernel $K_{l}$; and $s_{l}$ is the stride of the convolution kernel $K_{l}$. The depthwise convolution with a filter in each input channel (input depth) can be written as:

(1) $$D_{l}^{h_{l-1}^{c},d_{l}^{h},d_{l}^{w}}=\left(H_{l-1}^{h_{l-1}^{c}}⊗K_{l}^{h_{l-1}^{c}}\right)\left(d_{l}^{h},d_{l}^{w}\right),\;=\sum_{k^{dh}=1}^{K^{dh}}\sum_{k^{dw}=1}^{K^{dw}}H_{l}^{h_{l-1}^{c},s_{l}\cdot d_{l}^{h}+k^{dh},s_{l}\cdot d_{l}^{w}+k^{dw}}K_{l}^{h_{l-1}^{c},k^{dh},k^{dw}}+a_{l}^{h_{l-1}^{c}},$$

where $D_{l}\in {\mathbb{R}}^{H_{l-1}^{c}\times D_{l}^{h}\times D_{l}^{w}}$ is the output of the first convolution on the l^th^ DWS convolution block. $D_{l}^{h}$ and$\;D_{l}^{w}$ are the height and width of $D_{l}$, respectively. Figure 2 shows the operation process of the first convolution in DWS convolution. 

Next, $D_{l}$ is input into ReLU activation function after batch normalization (BN). The process is described as below:

(2) $$D_{l}^{\prime }=ReLU\left(BN\left(D_{l}\right)\right),\;$$

where *ReLU* is the non-linear activation function of linear rectification; *BN* is the batch normalization;$\;D_{l}^{\prime }\in {\mathbb{R}}^{H_{l-1}^{c},D_{l}^{h},D_{l}^{w}}$ is the output of ReLU. The second input of DWS convolution is $D_{l}^{\prime }$. 

**Figure 3:**
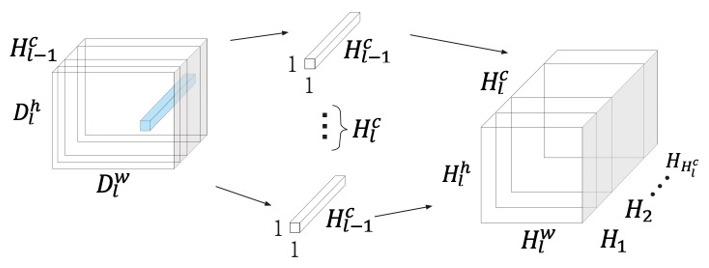
The second step of depthwise separable convolution: learning cross-channel information using $H_{l}^{c}$ different kernels $Z_{l}$.

In addition, $H_{l}^{c}$ number of 1 × 1 convolution kernels $Z_{l}\in {\mathbb{R}}^{H_{l-1}^{c}}$ and a bias vector $b_{l}\in {\mathbb{R}}^{H_{l}^{c}}$ are used to learn cross-channel information, with the process as follows:

(3) $$S_{l}^{h_{l}^{c},d_{l}^{h},d_{l}^{w}}=\left(D_{l}^{\prime h_{l-1}^{c}}⊗Z_{l}^{h_{l}^{c}}\right)\left(d_{l}^{h},d_{l}^{w}\right)\;=\sum_{h_{l-1}^{c}=1}^{H_{l-1}^{c}}D_{l}^{\prime h_{l-1}^{c},d_{l}^{h},d_{l}^{w}}Z_{l}^{h_{l}^{c},h_{l-1}^{c}}+b_{l}^{h_{l}^{c}},$$

where $S_{l}\in {\mathbb{R}}^{H_{l}^{c},D_{l}^{h},D_{l}^{w}}$ is the output of the second convolution block on the l^th^ DWS convolution. Figure 3 shows the operation process of the second convolution in DWS convolution. 

The down-sampling is used on the application feature dimension after each $H_{l}$ of the DWS convolution block, for example, maximum pooling. In Equations (1) and (3), $O(H_{l-1}^{c}\cdot K_{l}^{dh}\cdot K_{l}^{dw}\cdot D_{l}^{h}\cdot D_{l}^{w}+H_{l}^{c}\cdot H_{l-1}^{c}\cdot D_{l}^{h}\cdot D_{l}^{w})$ and $H_{l-1}^{c}\cdot K_{l}^{dh}\cdot K_{l}^{dw}+H_{l-1}^{c}\cdot H_{l}^{c}$ are the computation complexity and the number of parameters (neglecting deviation), respectively. Therefore, the computational complexity and the number of parameters are ${\left(H_{l}^{c}\right)}^{-1}+{\left(K_{l}^{dh}\cdot K_{l}^{dw}\right)}^{-1}$ times lower than a standard convolution operation with the same functions. The final output of the DWS convolution block $H_{L}\in {\mathbb{R}}^{H_{L}^{c}\times H_{L}^{h}\times H_{L}^{w}}$ is compressed into one-dimensional vector $H^{\prime }\in {\mathbb{R}}^{F}$, where $H^{\prime }$ is a one-dimensional feature vector that is extracted by learnable feature extractor inspired by auditory perception and F is the length of the feature vector.

### 3.3. Time-Dilated Convolution

Dilated convolutions introduce a new parameter called “dilation rate” to the convolutional layer, which defines the spacing of values when the convolutional kernel processes data. The purpose of this structure is to provide a larger receptive field without the pooling layer (pooling layer will lead to information loss) and with the same amount of computation. Since the dilated convolutions can cluster and learn multi-scale information, they are widely used in the visual field of deep learning now and have achieved outstanding performances in target detection and image segmentation [35]. In addition, the feature extractor obtains the one-dimensional underwater acoustic feature vector $H^{\prime }\in {\mathbb{R}}^{F}$ at continuous *T* times, then these $H^{\prime }$ into the two-dimensional matrix $I\in {\mathbb{R}}^{T\times F}$ in the fusion layer, where T and F respectively represent the height and width of the matrix, and I is the input of the two-dimensional time-dilated convolution. We use the two-dimensional time-dilated convolution to create a language model of the input, and the classifier is the linear layer of the Softmax activation function.

**Figure 4:**
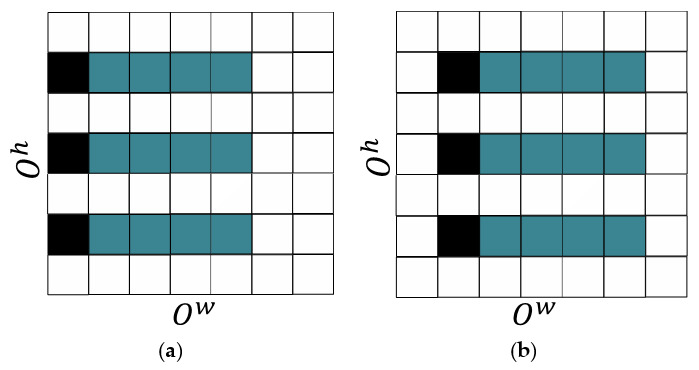
The illustration of the process described in Equation (4) using $\xi^{h}=2$ and processing two consecutive patches of O. Squares coloured with cyan signify the elements participating at the processing of $O^{o_{l}^{h},o_{l}^{w}}$, and coloured with grey are the elements of $O^{o_{l}^{h},o_{l}^{w}+1}$. (**a**) Processing of $O^{o_{l}^{h},o_{l}^{w}}$; (**b**) Processing of $O^{o_{l}^{h},o_{l}^{w}+1}$.

The time-dilated convolution network here used for long-term mode consists of J time-dilated convolution blocks. The j^th^ time dilated convolution block obtains the output of the previous block as input, i.e., $O_{j-1}\in {\mathbb{R}}^{O_{j-1}^{c}\times O_{j-1}^{h}\times O_{j-1}^{w}}$, where $O_{j-1}^{c}$, $O_{j-1}^{h}$ and $O_{j-1}^{w}$ are channel number, height and width output by the j−1^th^ time-dilated convolution block, respectively. $O_{0}=I$ is the feature matrix of the input ship-radiated noise, and $O_{0}^{c}=1$, $O_{0}^{h}=T$, $O_{0}^{w}=F$. The output of the jth time-dilated convolution block is $O_{j}\in {\mathbb{R}}^{O_{j}^{c}\times O_{j}^{h}\times O_{j}^{w}}$, where $O_{j}^{c}$, $O_{j}^{h}$ and $O_{j}^{w}$ are channel number, height and width of the jth time-dilated convolution block, respectively. The jth time-dilated convolution consists of $O_{j}^{c}$ kernels $K_{j}^{\prime }\in {\mathbb{R}}^{O_{j-1}^{c}\times K_{j}^{\prime h}\times K_{j}^{\prime w}}$ and bias vector $b_{j}^{\prime }\in {\mathbb{R}}^{O_{j}^{c}}$, where $K_{l}^{dh}$ and $K_{l}^{dw}$ are the height and width of the kernel $K_{j}^{\prime }$, respectively. Thus we have:

(4) $$Q_{j}^{o_{j}^{c},o_{l}^{h},o_{l}^{w}}=\left(O_{j-1}^{o_{l-1}^{c}}⊗K_{j}^{\prime o_{l-1}^{c}}\right)\left(o_{l}^{h},o_{l}^{w}\right)\;=\sum_{o_{j-1}^{c}=1}^{O_{j-1}^{c}}\sum_{k^{dh}=1}^{K^{dh}}\sum_{k^{dw}=1}^{K^{dw}}O_{j-1}^{o_{j}^{c},o_{l}^{h}+\xi^{h}\cdot k^{dh},o_{l}^{w}+k^{dw}}K_{j}^{\prime o_{j}^{c},o_{j-1}^{c},k^{dh},k^{dw}}+b_{j}^{\prime o_{j}^{c}},\;$$

where $\xi^{h}$ is the dilation rate of $K_{j}^{\prime }$ at $K_{j}^{\prime h}$ dimension. It should be noted that dilation is conducted only in the time dimension in this paper. The dilation rate $\xi^{h}$ multiplied by $k^{dh}$ is used for visiting the element of $O_{j-1}$.This allows context information to be clustered in proportion at the output of the operation [35]. In fact, this means that the feature result calculated with the time-dilated convolution is calculated from a larger area. Consequently, a longer time context can be used to create the recognition model. The process described in Equation (4) is shown in Figure 4. 

Next, $Q_{j}$ is input into the ReLU activation function after batch normalization (BN). The process is described as below:

(5) $$O_{j}=ReLU\left(BN\left(Q_{j}\right)\right).\;$$

In the last formula, ReLU is the non-linear activation function of the linear rectification; BN is branch normalization; $O_{j}\in {\mathbb{R}}^{O_{j}^{c},O_{j}^{h},O_{j}^{w}}$ is the output of ReLU. The output of final time-dilated convolution block $O_{J}\in {\mathbb{R}}^{O_{J}^{c},O_{J}^{h},O_{J}^{w}}$ is compressed into a one-dimensional vector $O^{\prime }\in {\mathbb{R}}^{O_{J}^{c}\times O_{J}^{h}\times O_{J}^{w}}$, then $O^{\prime }$ is input into the subsequent classifier $Cls\left(\cdot \right)$. The classification recognition for the underwater acoustic target is conducted as follows:

(6) $$\hat{Y}=Cls\left(O^{\prime }\right).$$

In Equation (6), $\hat{Y}=\;\left[{\hat{y}}_{1},\cdots ,{\hat{y}}_{C}\right]$ is the classification result predicted by the model for the underwater acoustic target. We use time-dilated convolution networks instead of RNN, which can effectively model long-term context, intra-class and inter-class activities for underwater acoustic target recognition. The model parameters are optimized through minimizing the cross-entropy loss between $\hat{Y}$ and Y.

## 4. Model Evaluation

In order to evaluate the proposed method, we used data samples from real civil ships, and used F1 score and precision as evaluation indexes. The artificially designed features, including waveform, wavelet, Mel-frequency cepstral coefficients (MFCC), Hilbert-Huang Transform (HHT), Mel frequency, non-linear auditory feature, spectral and cepstrum features are compared with those automatically extracted by the deep separable convolutional neural network. In addition, the histogram and (t-distributed stochastic neighbor embedding) t-SNE [45] are visualized the clustering performance of the proposed method. 

### 4.1. Dataset and Data Pre-Processing

**Figure 5:**
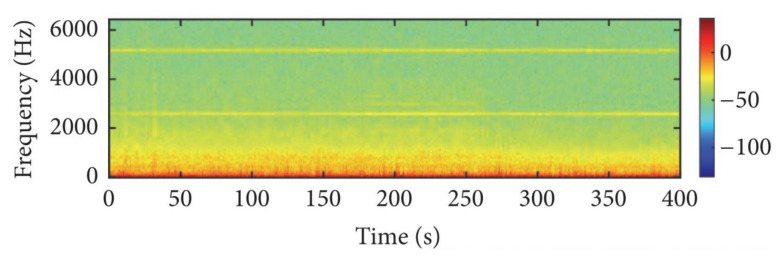
A frequency domain diagram of underwater noise.

**Figure 6:**
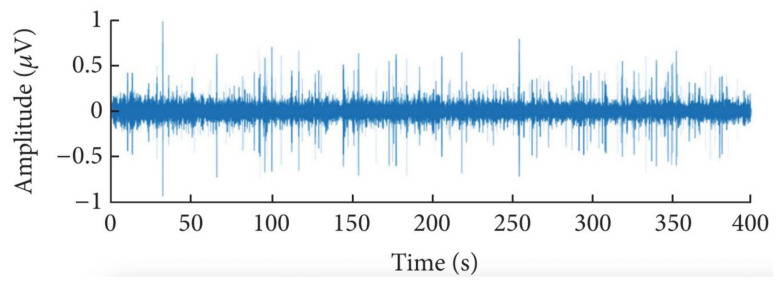
A time domain diagram of underwater noise.

The dataset contains small ship, big ship, and ferry. The data are sampled at anchorage ground, and the frequency is 48,000 Hz. In the experiment, 80% of samples of each class are used as a training set, while the remaining 20% of samples are used as a testing set. Figure 5 shows a frequency domain diagram of underwater noise. Figure 6 shows a time domain diagram of underwater noise. 

Each record is generated by a WAV (Waveform Audio File Format) audio file. The records include training dataset and testing dataset; 80% of samples of each class are used as the training set and the remaining 20% of samples of each class are used as the testing set. Each record is divided into an audio segment of 10 s, the sampling time of the training samples and test samples are 45 ms and the sampling interval is 12.5 ms. The network training and testing are performed on the raw time domain data without any preprocessing. The total time and number of each type of samples in the training data set and the test data set are shown in Table 1.

It can be seen from Table 1 that the number of samples of each class is seriously unbalanced. The sample number of small ships accounts for 55.7% of the total sample number, more than half the total sample number, while the big ship samples only account for 11.9% of the total sample number. From the sample distribution of each class, the number of the category sample size is very uneven. For a different classification sample set, the F1 score is a better index than accuracy. To evaluate the performance of this model, we should not only look at the precision index but also look at the performance of classification and recognition. In this paper, F1 score and accuracy are adopted as the evaluation indexes.

### 4.2. Hyper-Parameters, Indexes and Evaluation Process

In order to evaluate the proposed method, this paper uses the dataset of real time-domain radiated noise, including three classes (*C* = 3), i.e., “small ship”, “big ship” and “ferry”. The sound fragments are divided into *T* = 800 vector sequences *X* of length *N* = 2176, and using a hamming window function with 75% overlap in the successive windows of 45 ms: first of all, doing the normalize to all of the input vectors of *X* sequence, then inputting *X* into the learnable feature extractor which outputs a radiated noise feature inspired by auditory perception; the length of feature vector is *F* = 100.

**Figure 7:**
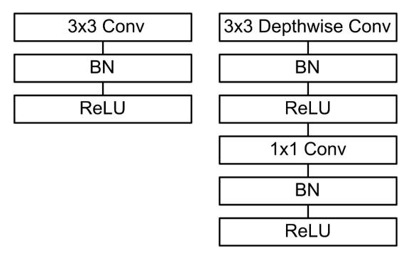
The depthwise separable convolutions with depthwise and pointwise layers followed by batch normalization (BN) and rectified linear unit (ReLU).

Table 2 lists the structure of the learnable feature extractor. The learnable feature extractor is built based on the above-mentioned one-dimensional DWS convolution, but the first layer is the standard one-dimensional convolution. All convolutions are followed by BN and ReLU activation functions. Figure 7 shows the comparison results between the standard convolution calculation process and the deep separation convolution calculation process.

The feature extractor network ultimately reduces the spatial resolution to 1. Our model structure puts a lot of computation into a 1 × 1 dense convolution, which can be achieved with a highly optimized Generic Matrix Multiplication (GEMM) function. However, an initial reordering called IM2COL is required in the memory to map it to GEMM. For example, this method is used in the popular Caffe package [46]. One by one convolution does not require reordering in memory; among all the deep separation convolution layers of the feature extractor network, 91% of the calculation time is spent in the 1 × 1 convolution, while 88% of the parameters are used; the computational complexity and number of parameters of each layer of deep separation convolution are shown in Table 3.

The structure of time-dilated convolutions inspired by the language model is shown in Table 4. The feature vector of one-dimensional underwater acoustic $H^{\prime }\in {\mathbb{R}}^{F}$ of *T* time is combined to form the two-dimensional matrix $I\in {\mathbb{R}}^{T\times F}$ (where, T=800 & F=100) and I is a time-dilated convolution network inspired by the language model. The convolution layers are followed by BN and ReLU activation function, but the pooling layer is not provided with non-linear activation function. The final pooling layer is input into softmax layer for classification. There are five layers in the time-dilated convolution network.

In order to evaluate the performance of the proposed method, we use F1 score and accuracy as the indexes of evaluation. We compare the recognition model of artificially designed features, a one-dimensional depthwise convolution network [23] without time-dilated convolution and the proposed depthwise separable convolutional neural networks. These artificially designed features include waveform, wavelet, MFCC, HHT, Mel frequency, non-linear auditory feature, spectrum and cepstrum. The deep separation convolutional neural network model is implemented on the framework of MXNET, A flexible and efficient library for deep learning [47]. The MXNET Python library runs on a nvidia RTX graphic card, and an asynchronous gradient similar to Inception V3 [43] is used to decline the optimizer RMSProp [48]. Table 5 lists the hyper-parameters of the proposed model.

Some researchers of neurosciences found that the brain can change its structure and functions to meet learning demands. In contrast to the large models of training, we use the techniques of less regularization and data processing, because the small models are not easy to overfit. Driven by the time domain signal of ship radiated noise, all parameters of the depthwise separable convolutional neural network are learned from the actual data. The frequency decomposition and perception ability of depthwise separable convolution networks are also learnable and adjustable.

## 5. Results and Discussion

The configuration of the server running the neural network is as follows: 64-bit Ubuntu 16.04 operating system, 64 GB memory, 52 CPU kernels and equipped with a TITAN RTX GPU accelerated computing card from NVIDIA (Computer systems design services company).

**Figure 8:**
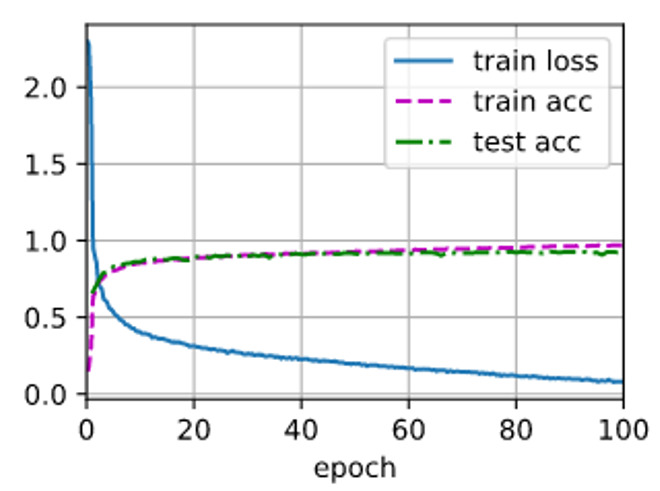
The training process of the model.

In this paper, the original time-domain ship-radiated noise data are used to train and test the model. The training parameters are 100 iterations, the size of the training batch is 800 and the training rate is 0.9. The detailed training process is shown in Figure 8:

As shown in Figure 8, in the process of model training, there is no over-fitting or under-fitting phenomenon, and there is no gradient disappearance or gradient explosion. By using the model with measured data, the final training result is that the recognition accuracy of the training data and the test data is 95.9% and 90.9%, respectively, which shows that the model has a high recognition accuracy. 

**Figure 9:**
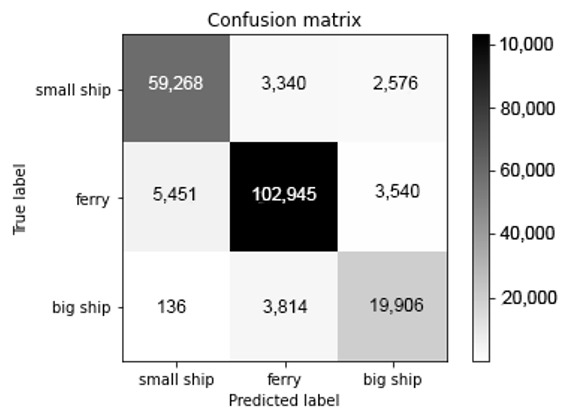
The confusion matrix of the proposed model obtained from testing data.

With 90.9% recognition accuracy, the model works well for underwater acoustic data with strong noise. Since the number of samples of each class varies greatly, we provide a confusion matrix for the recognition result of the proposed model, as shown in Figure 9. Each row of the confusion matrix corresponds to the real label and each column corresponds to the predicted label. 

Figure 9 shows that the recognition results are very stable among the classes, indicating that the model has good recognition stability. 

In order to more comprehensively evaluate the recognition performance of this method, on the basis of reflecting the recognition accuracy of the overall classification performance index of the model, the *F1*-score index reflecting the recognition performance of each class of the model is added. The *F1-*score for each class is calculated from a harmonic average of the accuracy and recall rates for that class, which is a better measure than accuracy for unbalanced datasets because both accuracy and recall rates are taken into account. The *F1*-scores for each class are “weighted” average and “micro” average. The accuracy rate, recall rate and *F1*-score of each category calculated by the recognition results in this paper are shown in Table 6:

As shown in Table 6, the *F1* score of this model is very good, indicating that this model has good classification accuracy and stability. The results of small boat class and ferry class are better, with *F1* score of 0.91 and 0.93 respectively. The worst results are for the big ship class, with an accuracy of 0.76, a recall rate of 0.83, and an *F1* score of 0.80. It could be that the mechanical systems of the boats are similar to those of ferries, or some boats were passing by during the collection of ferry samples.

**Figure 10:**
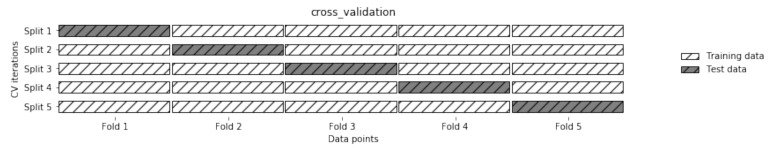
The whole process of 5-fold cross validation.

**Figure 11:**
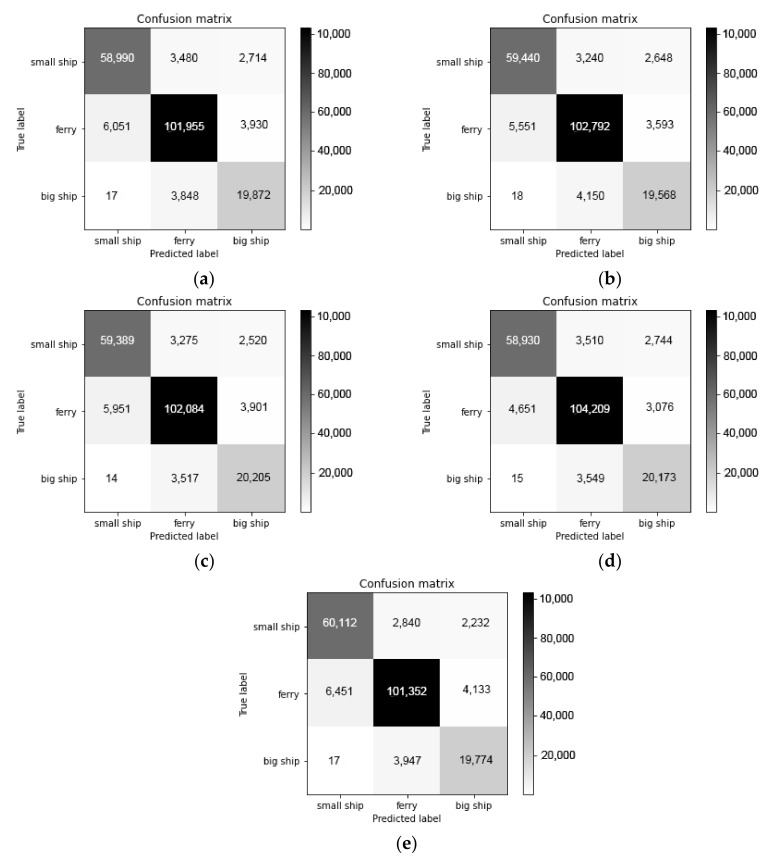
The confusion matrix of 5-fold cross validation: (**a**) fold 1; (**b**) fold 2; (**c**) fold 3; (**d**) fold 4; (**e**) fold 5.

In order to verify the non-unfitting and non-overfitting of the model, the *k*-fold cross-validation method is used. Cross validation is a statistical method to evaluate the generalization performance, which is more stable and comprehensive than the method of single partition of training set and test set. The data are divided many times and many models need to be trained. The most common cross validation is *k*-fold cross validation, where *k* is the number specified by the user. In this paper, we set *k = 5*. When we perform a 5-fold cross validation, the data are first divided into five equal parts, each of which is called a fold. The first fold is used as the test set, and the other folds are used as the training set to train the first model. The model is constructed with 2~5 trade-off data, then the accuracy is evaluated on 1 trade-off. Then another model is built, where we use 2-fold as the test set and others folds as the training set. For the five times of dividing the data into training set and test set, the accuracy should be calculated each time. Finally, we get five accuracy values. The whole process is shown in Figure 10, and the confusion matrix of 5-fold cross validation is shown in Figure 11. The validation results of 5-fold cross validation are listed in Table 7. 

From the experimental results of Table 7, the proposed model has good generalization performance, and there is no serious unfitting and overfitting. In order to simulate practical applications of recognition for ship-radiated noise, the classification accuracy of each acoustic event is used to measure the classification performance of the model, which is defined as the percentage of all acoustic events that are correctly classified. The classification accuracy of the proposed model and the comparison model is shown in Table 8.

HOS is high order statistics feature. MFCC is Mel-frequency cepstral coefficients. As shown in Table 8, compared with traditional underwater acoustic target recognition methods, the proposed model effectively improves the classification accuracy of the underwater acoustic target. Due to the complexity of the marine environment, it is also very important to improve the generalization performance and reduce the complexity of the model. Therefore, the regularization term of the first-order norm is added in the training process of the model to ensure good generalization performance of the model at the appropriate sacrifice of training classification accuracy. When the regularization term of the first-order norm is added, the classification accuracy of this model is 90.9%, which is 6.8% higher than that of the traditional recognition model, which is 85.1%, indicating that the classification recognition model significantly improves the classification accuracy of the traditional recognition method.

**Figure 12:**
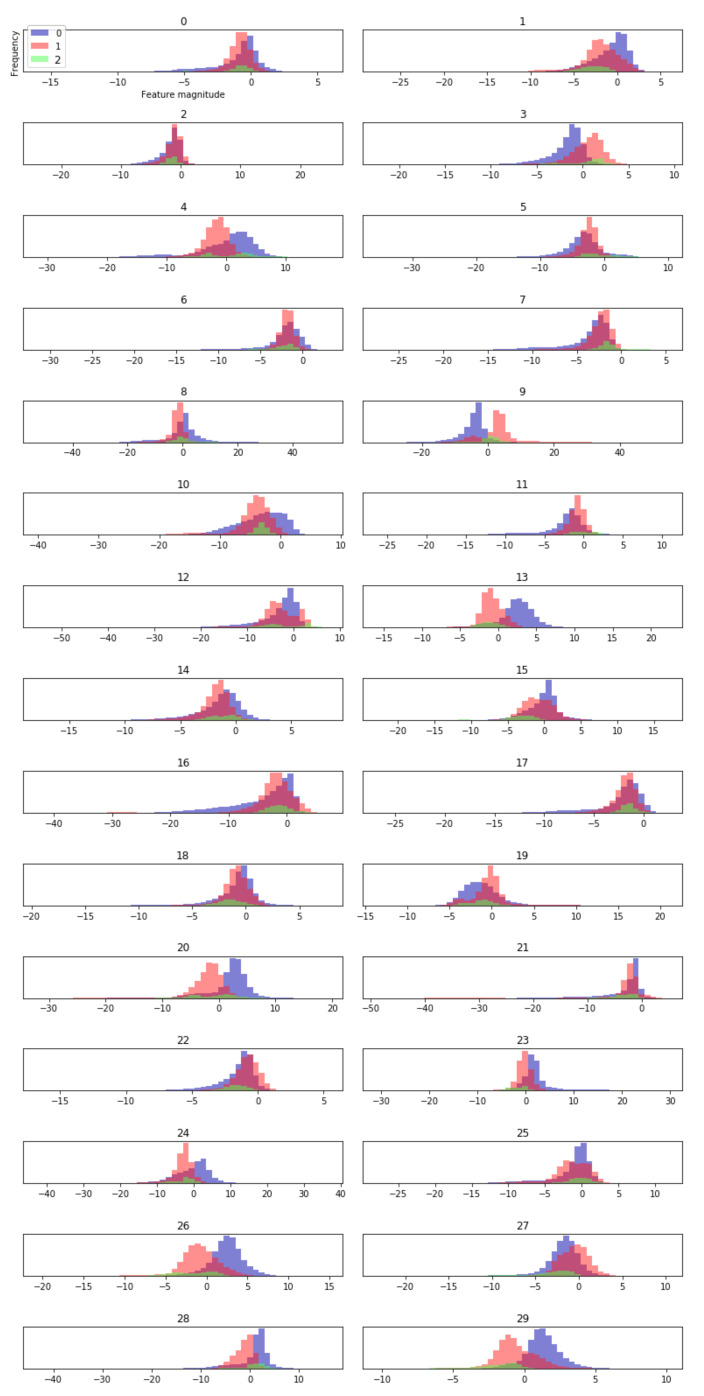
The histogram of features learned from the proposed model: 0 is small ship, 1 is big ship, 2 is ferry.

Next, the distribution of features extracted by the model is analyzed by visualization method. Here, a histogram is created for each extracted dimension feature, and the occurrence frequency (called bin) of the data points of a one-dimension feature in each class is calculated. This allows us to understand the distribution of each dimension’s features in each class and how the eigenvalues are different between classes. In this paper, 100-dimensional features are extracted from underwater acoustic targets. Figure 12 is part of the histogram of feature results extracted from the model. As shown in Figure 11, the feature vectors extracted by the proposed model for all training samples have obvious distribution differences among different classes, while the feature distributions within the same class are stable and consistent, which indicates that the method of feature extraction by the proposed model is effective for classification.

**Figure 13:**
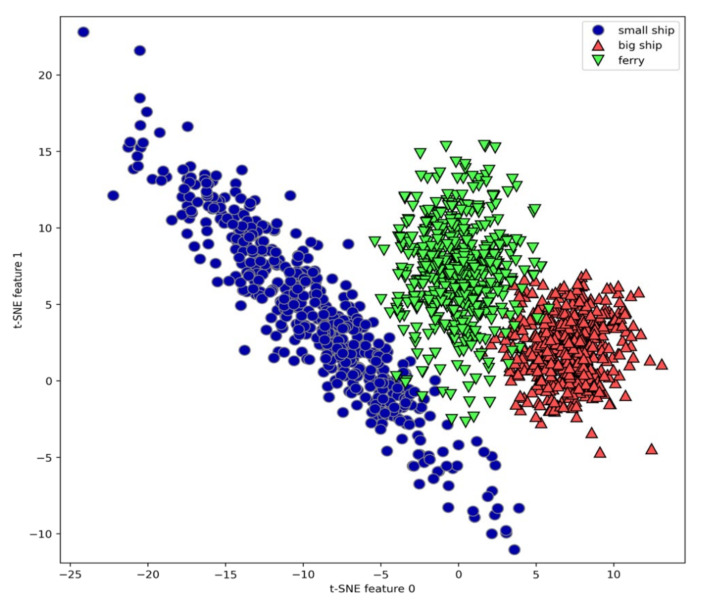
The scatter plot of features learned from proposed model using two components found by t-distributed stochastic neighbor embedding (t-SNE).

The manifold learning algorithm is used to carry out complex mapping of 100-dimensional feature vectors extracted from all samples, and a good visual 2-dimensional vector is obtained. We use the t-SNE algorithm to visualize the feature vectors of underwater acoustic targets. Figure 13 shows the scatter plot of the two-dimensional vector obtained from the complex mapping of 100 feature vectors. Here, we use the corresponding number of each class as a symbol to show the position of each class. It can be seen that each class is relatively well separated, indicating that the feature results extracted by the model have a good clustering effect, and visually proving that the feature extracted by the underwater acoustic target has good separability and stability.

## 6. Conclusions 

In this paper, a new depthwise separable convolutional neural network is proposed to identify ship radiated noise from original time-domain waveforms in an end-to-end mode. The deep features containing the internal information of the target are extracted by a DWS convolution network, which reflects the deep acoustic information extraction structure of the auditory system. By convolution decomposition of different frequency components of ship-radiated noise, the frequency distribution characteristics of ship radiated noise are revealed. The time-dilated convolution is used for modeling long time contexts, which can make full use of the intra-class and inter-class information for underwater acoustic target recognition just like the language model. Inspired by the plasticity neural mechanism, all the parameters in the model are learned and optimized under the drive of the time-domain ship radiated noise, so as to accomplish the underwater acoustic target recognition task. The average classification recognition rate reaches 90.9% when tested on a real civil ship acoustic signal set. Although the recognition rate is high, there is still a certain gap between it and the practical application, and the recognition rate needs to be further improved. The experimental results also show that the extracted 100-dimensional features of underwater acoustic target have good separability and stability, and the deep learning method based on auditory perception has great potential in improving the classification performance of underwater acoustic target recognition. 

## Figures and Tables

**Table 1:** The experimental data description.

Data Set	Class	No. Segments	Total Time (Hour)	No. Samples	Percentage
Training	small ship	326	0.91	260,736	25.9%
	ferry	560	1.55	447,744	44.6%
	big ship	119	0.33	95,424	9.5%
Test	small ship	81	0.23	65,184	6.5%
	ferry	140	0.39	111,936	11.1%
	big ship	30	0.08	23,856	2.4%

**Table 2:** The structure of the learnable feature extractor.

Type	Stride	Filter Shape	Input Size
Conv1D	50	204 × 1 × 64 dw	2176 × 1
Conv1D dw	2	12 × 64 dw	40 × 64
Conv1D	1	1 × 1 × 64 × 128	15 × 64
Conv1D dw	1	15 × 128 dw	15 × 128
Conv1D	1	1 × 1 × 128 × 100	1 × 100

**Table 3:** The resource per depthwise separable layer.

Type	Mult-Adds	Parameters
Conv1D dw	11,520	832
Conv1D	122,880	8320
Conv1D dw	1920	2048
Conv1D	12,800	12,900

**Table 4:** The structure of the time-dilated convolutions.

Type	Stride	Dilation	Filter Shape	Input Size
Conv2D Dilation	1 × 1	12 × 1	3 × 3 × 1 × 64	800 × 100 × 1
Max Pool	2 × 2	1 × 1	Pool 2 × 2	776 × 98 × 64
Conv2D Dilation	1 × 1	12 × 1	3 × 3 × 64 × 128	388 × 49 × 64
Max Pool	2 × 2	1 × 1	Pool 2 × 2	364 × 47 × 128
Conv2D Dilation	1 × 1	12 × 1	3 × 3 × 128 × 256	182 × 23 × 128
Avg Pool	2 × 2	1 × 1	Pool 2 × 2	158 × 21 × 256
Conv2D Dilation	1 × 1	12 × 1	3 × 3 × 256 × 512	79 × 10 × 256
Avg Pool	2 × 2	1 × 1	Pool 2 × 2	55 × 8 × 512
Conv2D Dilation	1 × 1	12 × 1	3 × 3 × 512 × 512	27 × 4 × 512
Avg Pool	2 × 2	1 × 1	Pool 2 × 2	3 × 2 × 512
Softmax			Classifier	1 × 1 × 3

**Table 5:** The hyper-parameters of the proposed model.

Parameters	Values
Learning Rate	0.001
Batchsize	800
Epochs	100
Optimizer	RMSprop

**Table 6:** The precision, recall and *F1* score for each class.

Class	Precision	Recall	*F1* Score	Support
small ship	0.91	0.91	0.91	65,184
ferry	0.94	0.92	0.93	111,936
big ship	0.76	0.83	0.80	23,856
Accuracy			0.91	
Macro avg	0.87	0.89	0.88	
Weighted	0.91	0.91	0.91	

**Table 7:** The results of 5-fold cross validation.

Fold	Weighted Average *F1* Value
1	0.90
2	0.91
3	0.91
4	0.92
5	0.90

**Table 8:** The classification results of proposed model and compared models.

Input	Methods	Accuracy
HOS [49]	Support vector machine (SVM)	85.1%
Waveform [9,50]	SVM	78.9%
Wavelet [11]	SVM	84.3%
MFCC [51]	SVM	79.1%
Mel-frequency	SVM	84.6
Nolinear auditory	SVM	86.7
Spectral [27,52]	Deep neural network (DNN) [53]	87.0%
Cepstral [10,54]	DNN [44]	86.9%
Raw time domain data	Convoluted neural network (CNN) model [25]	88.4%
Raw time domain data	Convolution recursive neural network (CRNN) model [28]	89.2%
Raw time domain data	Proposed model	90.1%
